# An Informationally Structured Room for Robotic Assistance ^†^

**DOI:** 10.3390/s150409438

**Published:** 2015-04-22

**Authors:** Tokuo Tsuji, Oscar Martinez Mozos, Hyunuk Chae, Yoonseok Pyo, Kazuya Kusaka, Tsutomu Hasegawa, Ken'ichi Morooka, Ryo Kurazume

**Affiliations:** 1 Faculty of Information Science and Electrical Engineering, Kyushu University, Fukuoka 819-0395, Japan; E-Mails: tsuji@ait.kyushu-u.ac.jp (T.T.); huchae@irvs.ait.kyushu-u.ac.jp (H.C.); pyo@irvs.ait.kyushu-u.ac.jp (Y.P.); kusaka@irvs.ait.kyushu-u.ac.jp (K.K.); morooka@ait.kyushu-u.ac.jp (K.M.); kurazume@ait.kyushu-u.ac.jp (R.K.); 2 School of Computer Science, University of Lincoln, Lincoln LN57PN, UK; E-Mail: omozos@lincoln.ac.uk; 3 Kumamoto National College of Technology, Kumamoto 861-1102, Japan; E-Mail: hasegawa@kumamoto-nct.ac.jp

**Keywords:** assistive technologies, intelligent sensors, service robotics, smart home, ambient intelligence, distributed sensors

## Abstract

The application of assistive technologies for elderly people is one of the most promising and interesting scenarios for intelligent technologies in the present and near future. Moreover, the improvement of the quality of life for the elderly is one of the first priorities in modern countries and societies. In this work, we present an informationally structured room that is aimed at supporting the daily life activities of elderly people. This room integrates different sensor modalities in a natural and non-invasive way inside the environment. The information gathered by the sensors is processed and sent to a centralized management system, which makes it available to a service robot assisting the people. One important restriction of our intelligent room is reducing as much as possible any interference with daily activities. Finally, this paper presents several experiments and situations using our intelligent environment in cooperation with our service robot.

## 1. Introduction

Inside the many applications related to quality of life technologies, elderly care is one of the most promising ones, both in social and economic terms. Improving the quality of life of the elderly is also one of the first priorities in modern countries and societies, where the percentage of elderly people is rapidly increasing due mainly to great improvements in medicine during the last few decades.

A potential approach to support daily life activities of elderly people consists of creating a living environment supported by different technologies, including different sensor modalities and service robots. A common idea in assistive environments consists of gathering information about people's activities together with their surroundings in a way that an intelligent decision system can support the persons [1–7]. In addition to intelligent environments, service robots can be available to assist people in their daily activities and environments [8–12]. Actually, it is expected that service robots will soon be playing the role of companion for elderly people or as general assistants for humans with special needs at home. However, human environments are very complex and sometimes difficult to monitor only with the sensors mounted on the robot. One solution to this problem consists of supporting the service robot with information about the environment using ambient intelligent technologies [13–17].

**Figure 1:**
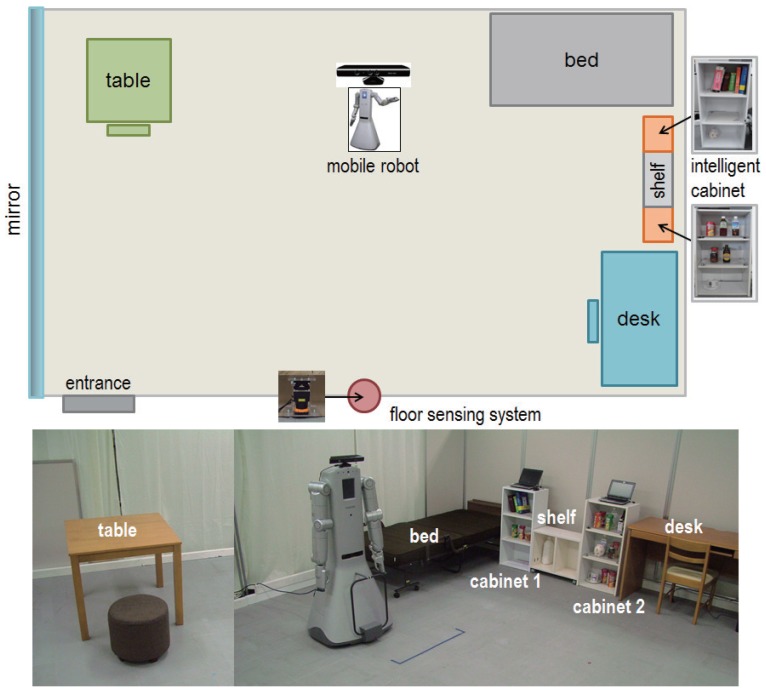
The top image outlines a map of our intelligent room. Pictures of different parts of the room are shown in the bottom images.

This intelligent room is composed of a network of distributed sensors that are installed at different locations, pieces of furniture and objects inside the room. These sensors monitor people's activity inside the room and send the information to a centralized management system, which processes the data and makes it available to a service robot that assists the inhabitants. In addition, the service robot can send information coming from its own perception system. Figure 1 shows an overview of our intelligent room together with our service robot. One important restriction in our intelligent environment is to avoid interfering with the daily activity of people and to reduce as much as possible the invasion of their privacy. In addition, we constrain the use of the camera on the robot to predetermined situations only. Finally, the sensors of our environment are weak in the sense that they acquire only a small part of the information needed for activity monitoring. Therefore, an important aspect of our informationally structured environment consists of the integration of the information provided by the weak sensors in order to determine changes in the environment.

The first ideas for our structured room appeared in [13,14] inside the Robot Town project, which established the main ideas and foundations to create a complete structured city. Our work extends the ideas from [13,14] and makes particular contributions to informationally structured rooms. The work in [15] also proposes an informative space where sensor data are managed using ubiquitous computing. However, the system in [15] only covers localization and navigation of the service robot. By contrast, in our work, we additionally integrate people activity and object detection and manipulation. An alternative paradigm is the physically-embedded intelligent system (PEIS) presented in [16], which combines the robot skills with ambient intelligence. PEIS provides a distributed environment that exchanges information among different functional software components. In our approach, in contrast, we use a centralized management system that provides the extra reasoning needed by the robot to support its tasks. In addition, implementations of the PEIS system usually rely on camera monitoring systems. However, we try to avoid vision systems in our approach to reduce as much as possible the invasion of privacy. Finally, strongly sensory structured micro-rooms are presented in [17]. These micro-rooms also aim to support elderly people, but the intelligent capabilities are limited, since the number of sensors is reduced to keep prices competitive in production. In comparison, our proposed structured room is composed of several different sensor modalities, including laser range finders and radio-frequency identification (RIF) systems. Moreover, the room is supported by a humanoid robot. Therefore, our system currently has a high cost. However, we think costs can be reduced in the future, since companies are lowering the prices of the sensors used. Moreover, the price of service robots is expected to lower as they reach mass production.

This paper extends our previous works in [7,13,18–22] with the following contributions:

1. Identified tracking of persons and movable furniture using an inertial sensor and a laser range finder.
2. Improved daily life object tracking by portable RFID tag readers and an RGB-D camera.
3. Robot service demonstration of the go-and-fetch task.

The rest of the paper is organized as follows. Section 2 describes the different sensor modalities that compose our informationally structured room. In Section 3, we describe our service robot. The centralized information system is described in Section 4. Finally, in Section 5, we present different experiments and scenarios for assistance and information updating in our room.

## 2. The Informationally Structured Room

In this section, we describe the different sensor modalities that are found in our informationally structured room. Our intelligent room represents a standard room in a house containing typical appliances and pieces of furniture, as shown in Figure 1. In particular, our room contains a bed, chairs, tables, shelves, cabinets and a service trolley. In the room, we can also find some typical commodities and objects, like, for example, cups, books, bottles or slippers. Some pieces of furniture and objects inside the room are equipped with sensors that help monitoring the activity inside the room. For example, the cabinets and the service trolley contain sensors to detect the objects that are situated on them. In addition, the service trolley and the wheel chair contain inertial sensors to facilitate their localization and activity monitoring. Finally, the room is equipped with a floor sensing system that tracks and detects people and objects moving inside the room. The sensors are distributed and situated in a way that they do not interfere with the daily activities of people living in the room. Moreover, we try to reduce as much as possible the invasion of people's privacy.

### 2.1. Intelligent Cabinets

**Figure 2:**
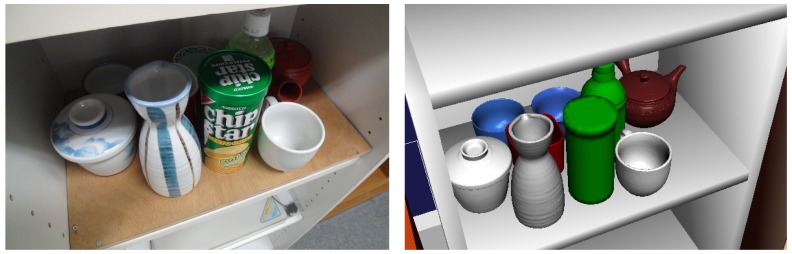
Information about objects provided by the intelligent cabinet. Image (**a**) shows the original position of the objects inside the intelligent cabinet; The image (**b**) shows the database viewer with their positions and the corresponding shape models.

Our informationally structured room contains two intelligent cabinets [19], as shown in Figure 1. Each of these cabinets is equipped with an RFID reader and load cells to detect the type and position of the commodities that are situated inside. Some of the commodities in our environment have an RFID tag attached that contains a unique identifier (ID) assigned to it. This ID is used to retrieve the attributes and characteristics of the commodity from the central database. The RFID readers can detect the presence of different commodities inside the cabinet. In addition, the information from the load cells is used to precisely localize the object inside the cabinet. Figure 2 shows one of the cabinets together with the information that it provides about some commodities that are found inside.

In this particular case, the intelligent cabinet contains nine objects that are detected and shown in our database viewer as in Figure 2. The objects inside the cabinet are difficult to detect with the vision sensor of the robot due to occlusions and difficult viewpoints. Thus, the intelligent cabinet helps the service robot by providing the position and measurements of the objects found inside. Moreover, information about the object ID, its position and weight with a time stamp are sent to our centralize database.

**Figure 3:**
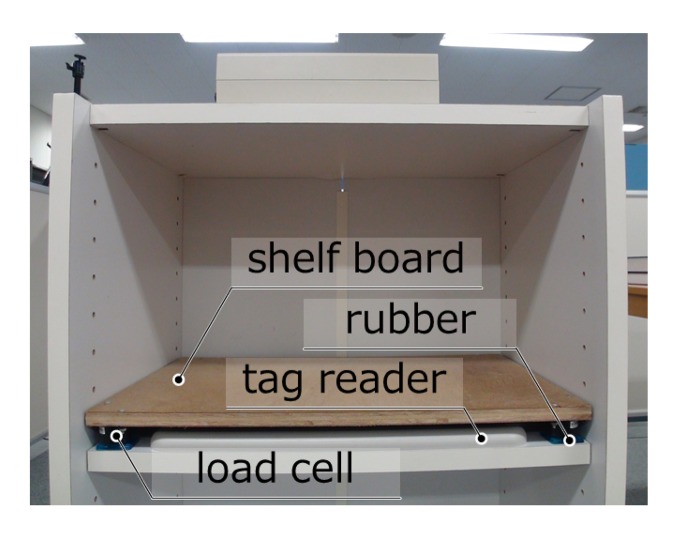
Structure of the intelligent cabinet.

The interval of inputting or outputting objects inside the cabinet has a limitation because of the convergence time of the material vibration of the sensor board. When an object is put on the board, the board vibrates, then if another object is put before the vibration converges, the position and weight of the objects will be inaccurate. To improve the response time and accuracy, we tested several materials for this system and found that plywood (with a 12-mm thickness) as the shelf board and seismic isolation rubber as the legs have good features for converging vibration (see Figure 3). We compare this new system to our our previous one based on acrylic boards [19]. The response time for the new system decreases to less than 400 m, while the previous system had a response time of about 600 ms. Even though the new position accuracy is slightly worse, the new error is inside acceptable ranges, as shown in Table 1.

### 2.2. Non-Static Intelligent Furniture

In addition to static pieces of furniture, our intelligent room contains some movable furniture and appliances. In particular, we include an intelligent service trolley and a wheelchair in the room.

The intelligent service trolley is equipped with the same sensor system as the intelligent cabinets, *i.e.*, one RFID reader and a load system composed of four load cells. In this way, the intelligent trolley can provide information about the objects that are on it at any time. The intelligent trolley is additionally equipped with one inertial sensor to help keep track of its position.

**Figure 4:**
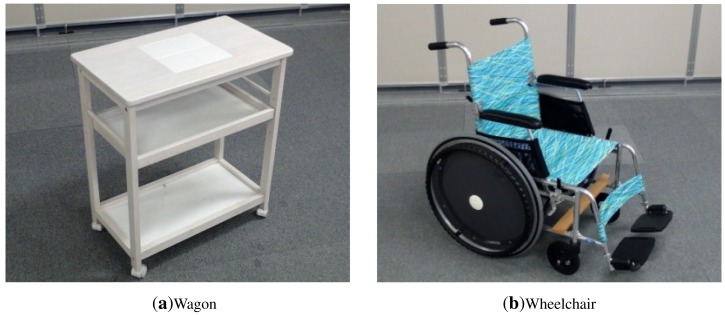
(**a**) shows the service trolley used in our room, while (**b**) depicts our wheelchair.

The wheelchair is also equipped with one inertial sensor to facilitate its localization inside the room. Figure 4 depicts images of our service trolley and wheelchair.

### 2.3. Floor Sensing System

**Figure 5:**
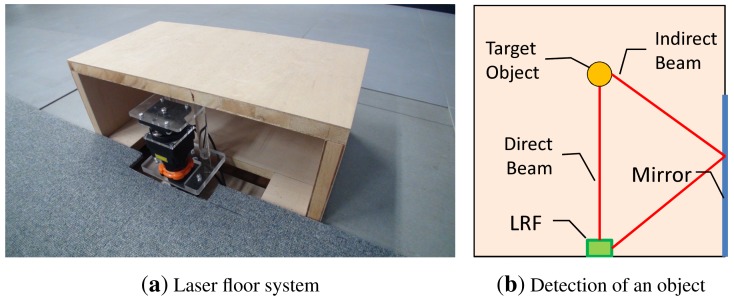
(**a**) shows the laser range finder used in our floor system; (**b**) depicts the configuration of the sensing system when detecting an object.

Our informationally structured room is further supported by a floor sensing system, which is used to detect moving objects and people. This system is composed of a laser range finder (LRF), which is situated on the floor on one side of the room, as depicted in Figure 1. The system is extended with a mirror located along one side of the room. This configuration reduces the number of dead angles for the LRF and increases the robustness against occlusions, as shown in Figure 5, thus improving the detection when clutter occurs [23].

The floor sensing system is able to detect the 2D blobs corresponding to different objects, people or robots. The system first applies static background subtraction and then extracts blobs in the rest of the measurements. However, the limited 2D information provided by the laser range finder is not enough to determine the particular entities moving around. Therefore, extra sensor modalities are used to help recognize particular moving objects and persons inside the room. Thus, we attach inertial sensors to the service trolley, the wheelchair and to the slippers of the habitants. By integrating inertial sensor information together with the 2D laser readings, we can locate and recognize each specific object/inhabitant. Details on tracking are given in the next section.

### 2.4. IMU-Based People Identification

Gate identification by attaching an inertial sensor to a person's waist is proposed in [24]. This method, however, requires attaching inertial sensors to persons or accessing their smart phones in their pockets.

**Figure 6:**
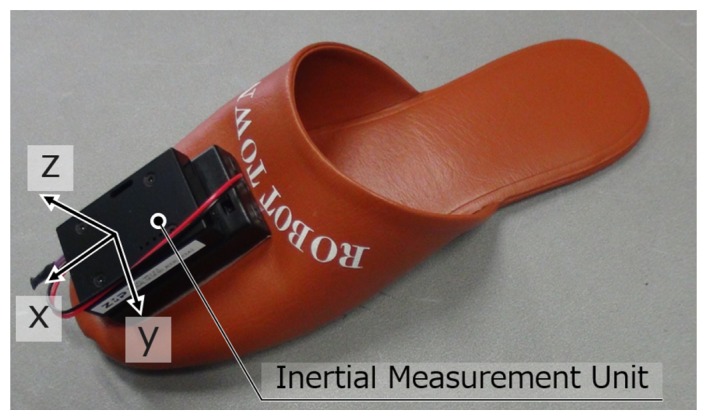
Inertial sensor attached to a slipper.

In this paper, we propose a method to identify different persons by attaching an IMU sensor to slippers as shown in Figure 6. Our method does not demand special settings for persons, and it is a natural way to identify visitors and track all persons in public buildings or at home. This is a very convenient means in Japan, because in this country, homes and some public spaces provide slippers for visitors. In this way, the activity of the habitant can be easily extracted, which is very useful for robot service and the life log of the habitant.

**Figure 7:**
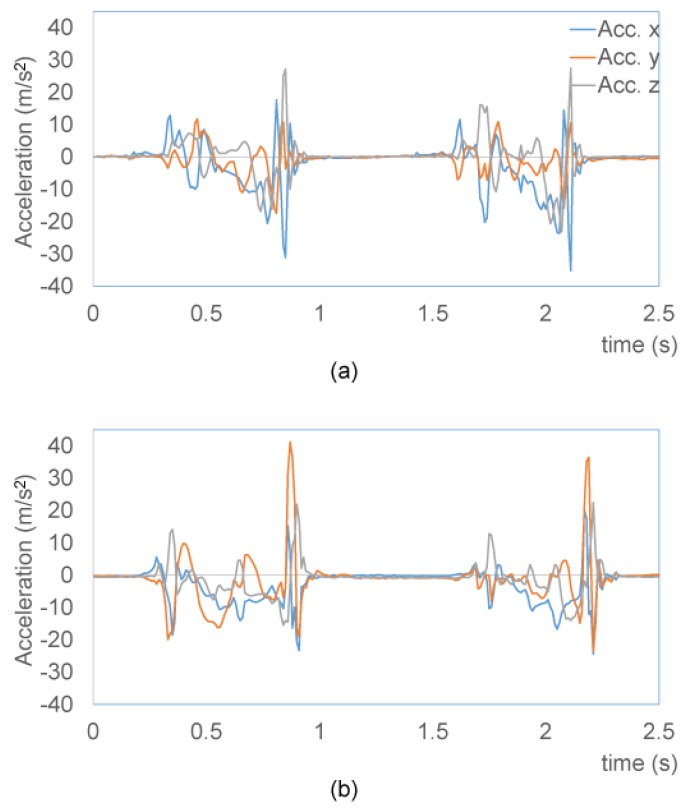
Foot acceleration for the first person (**a**) and second person (**b**).

Our system captures the acceleration and angle velocity of gait motion. The foot accelerations of two persons are shown in Figure 7. An autoregressive model [25] is applied to approximate the sequence of waves. The autoregressive model is expressed as:

(1) $${\text{x}}_{\text{t}}=\sum_{i=1}^{p}\Phi_{i}{\text{x}}_{\text{t}-\text{i}}+\in$$

where **x**_t_ = [*a_x_, a_y_, a_z_, w_x_, w_y_, w_z_*]*^T^* is the vector containing the three axis accelerations {*a_x_, a_y_, a_z_*} and angular velocities {*w_x_, w_y_, w_z_*}, {Φ_1_ ⋯ Φ*_p_*} are the parameters obtained using Yule-Walker equations [26] and *ϵ* is the modelling error. Finally, we set *p* = 8 in our experiments. The identification of the persons is then performed by finding the nearest-neighbour of the parameters {Φ_1_ ⋯ Φ*_p_*}.

### 2.5. People Tracking

In general, it becomes difficult to identify and track persons in a room with little invasion of people's privacy. To solve this problem, several methods have been proposed in the past. In [27,28], multiple persons are tracked using cameras. Some other works [29,30] also propose the combination of cameras and other sensors, such as the pressure sensors on the floor and acceleration sensors. However, it is still difficult to prevent invading persons' privacy if we use cameras.

Some other works avoid the use of cameras in order to keep privacy. The method presented in [31] uses LRF only and cannot identify specific persons. The tracking system in [32] is based on dead reckoning using an inertial sensor, but the accuracy decreases over time. The work in [33] uses passive RFID tags, but sometimes fails to track persons far from the tag reader. An ultrasonic tagging system [6] has a high cost, both in maintenance and space, since many large directional antennas are required for tracking the tags. The use of received signal strength indication (RSSI) of active RFID tags [34] and WiFi signals [35] is not accurate enough for localizing persons in a room.

**Figure 8:**
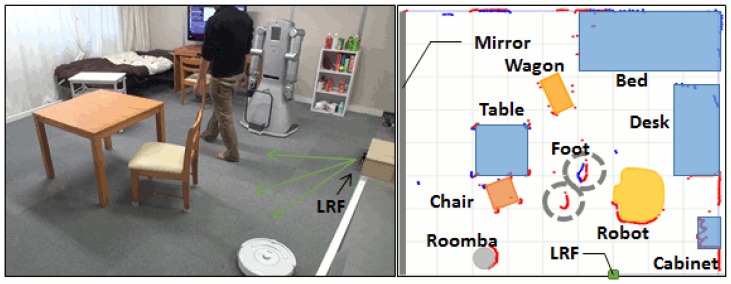
The right image depicts a 2D representation of the environment obtained using the laser range finder (LRF) where the person is marked with a circle. The left image shows the corresponding real scene.

In our system, people tracking is performed using the laser-based floor system by first applying static background subtraction and then extracting blobs in the rest of the measurements. An example detection of a person using the floor system is shown in the middle image of Figure 8.

**Figure 9:**
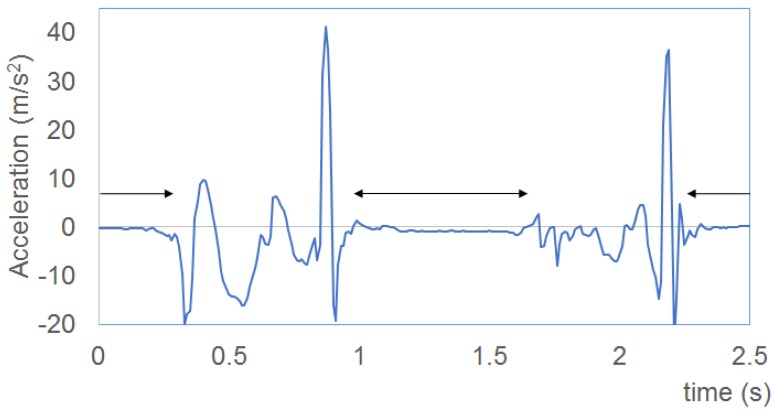
The acceleration (z-axis) of foot motion is shown. The double arrows indicate the period that the blobs are measured by the floor sensing system.

For multiple person tracking, we find the correspondence of blobs on the floor and inertial sensors. The foot repeats moving in the air and landing on the floor alternately. In the floor sensing data, the appearance and disappearance of blobs repeat, since the LRF is set only few centimetres above the floor and cannot measure the moving foot in the air. When the foot lands on the floor, a blob appears, and the inertial sensor of the foot detects the stopping of motion. When the foot leaves the floor, the blob disappears, and the inertial sensor detects the starting of motion. A blob and an inertial sensor correspond to each other by checking the timings (Figure 9).

### 2.6. Tracking of Non-Static Furniture

In the case of the service trolley and the wheelchair, we have to check all possible combinations of four candidate blobs that correspond to the wheels and match them with the geometrical restrictions of the specific object. In the case of the service trolley, the four wheels form a specific rectangle; and in the case of the wheelchair, they form a particular polygon.

The general procedure to characterize the different moving entities is as follows. For each laser observation, we first apply a static background subtraction and extract 2D blobs corresponding to the different moving entities inside the room. Each blob is assigned the nearest blob obtained in the previous measurement, thus obtaining a sequence of each blob position over time. Using these sequences, we can calculate the motion velocity for each blob. In addition, we obtain the velocity information of each moving entity provided by its inertial sensor. We then calculate the correlation between the velocity obtained by the inertial sensor and the velocity of each moving blob. High values in the correlation indicates that the blob is regarded as a candidate for the specific entity. Then, the entity is tracked as the centre of the blobs. For tracking occlusion robustly, the normal Kalman filter [36] with the state vector [*x, y, θ, ẋ, ẏ*]*^T^* is utilized, where (*x, y*), *θ* and (*ẋ, ẏ*) are the position, the direction and the velocity, respectively.

## 3. Service Robot

**Figure 10:**
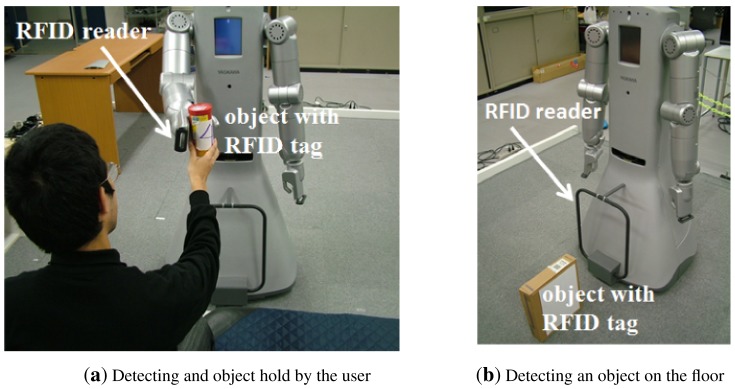
Two example situations where the robot is using its RFID sensors: (**a**) detecting and object hold by the user, and (**b**) detecting an object on the floor.

People inside our intelligent room are assisted by a SmartPal humanoid service robot from Yaskawa Electric Corporation (see Figure 10). This robot is responsible for fetching objects or pointing to them. The robot is composed of a mobile platform, two arms with seven joints and one-joint grippers used as hands. In addition, we equipped the robot with an RGB-D camera, which is activated for object recognition in restricted regions of interest and only under specific requests. In order to maintain the privacy of people, we do not use this camera for general vision purposes. Additional RFID readers are situated on the hands and front of the robot, as shown in Figure 10.

### 3.1. Visual Memory System

Our service robot is equipped with a visual memory system, which helps in the task of object searching and finding. The visual memory system is used by the robot to detect changes of predefined places where objects usually appear. In our case, we restrict the application of this visual system to the table of our intelligent room (see Figure 1). The reason for that is to retain the privacy of the people as much as possible and to avoid registering images of the user during his daily and private activities. The visual memory system is composed of two main steps. In the first one, changes are detected in the area of interest, the top of the table in our case, which usually corresponds to appearance, disappearance or movement of objects. In the second step, the areas corresponding to the changes are analysed, and new objects are categorized.

The first step of our visual memory is responsible for detecting changes in the area of interest, which is a table in our case. The change detector works as follows. At some point in time *t*_1_, the service robot takes a snapshot *z*_1_ of a table. Since we use an RGB-D camera, then our observation is composed of a 3D point cloud. At some later point in time *t*_2_, the robot takes a second snapshot *z*_2_ of the same table. The positions *p*_1_ and *p*_2_ of the robot during each observation are known and determined by our localization system, so that we can situate each observation in a global reference system. In addition, we improve the alignment of the two point clouds using the iterative closest point (ICP)algorithm. This step allows us to correct small errors that can occur in our localization system.

**Figure 11:**
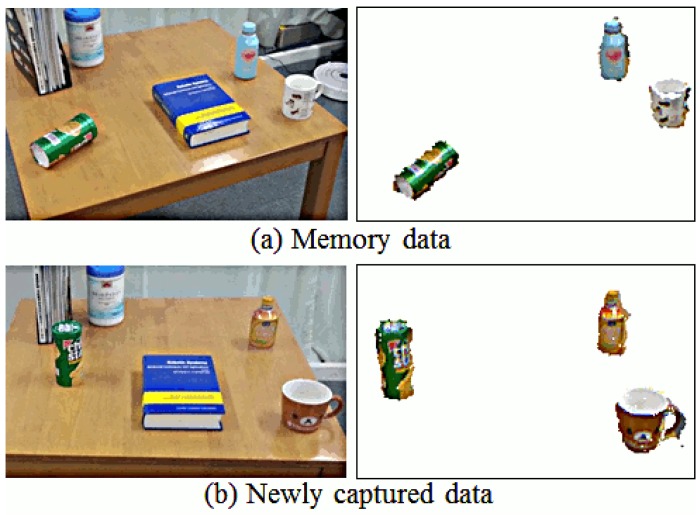
Changes detected between two consecutive views of a table. In (**a**) the first view is kept in memory; In (**b**) new detectd objects are shown.

For each independent view, we extract the plane corresponding to the top of the table by applying a method based on RANSAC. The remaining points, which pertain to the possible objects on top of the table, are projected to a 2D grid. The cells in this grid are clustered using connected components, and each resulting cluster is assumed to be the 2D representation of a different object on the table. We then compare the 2D clusters in each view and determine the different clusters between the two views, which correspond to changes on the table. The resulting change detection is shown in Figure 11.

### 3.2. Object Categorization

**Figure 12:**
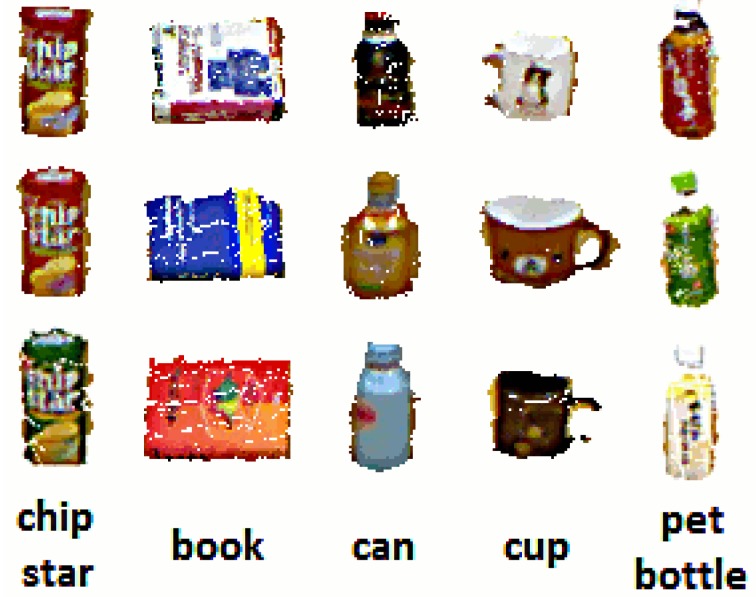
Dataset containing different types of daily life commodities.

**Figure 13:**
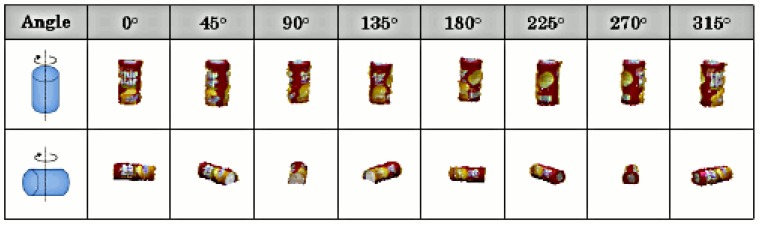
For each object in our dataset, we create example views at different orientations. The image shows an example for a Chipstar container.

The point clusters corresponding to possible changes on the table are further categorized into a set of predefined object categories contained in our database. This database contains typical commodities, as shown in Figure 12. We extend this dataset with different views of each object, as shown in Figure 13.

Our method finds the best matching between the point clusters representing possible objects and the objects in the database. Our matching method is based on correspondence grouping [37] using the signature of histograms of orientations (SHOT)3D surface descriptor [38]. In our experiments, we used two different approaches to find the best matching. In the first method, the best matching is obtained as the minimum distance according to:

(2) $$D=\frac{\text{corr}}{\max\left(N_{{\text{model}}_{j}},N_{\text{cluster}}\right)}$$

where *corr* represents the number of correspondences between keypoints in the model of our dataset and the keypoints in the cluster, *N_model_j__* indicates the number of keypoints found in the model and *N_model_* represents the number of keypoints found in the cluster.

The second method uses distance based on the centroid and standard deviation of each component in the descriptor for each cluster, as introduced in [39].

A comparison of object categorization results using different methods is presented in Section 5.

## 4. Town Management System

**Figure 14:**
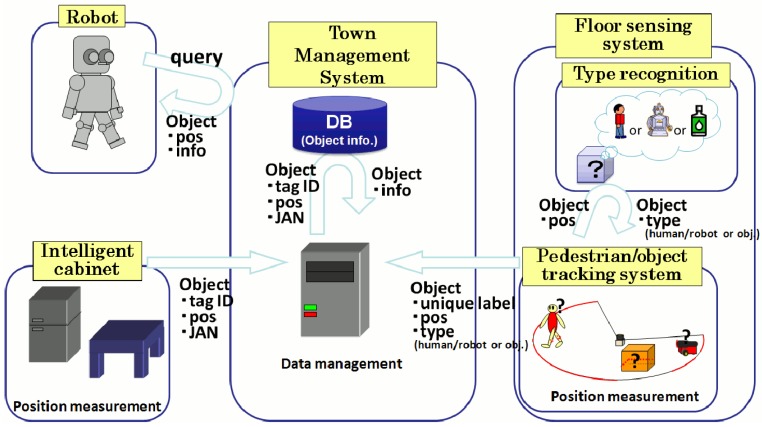
Information flow among the town management system (TMS) and the different sensor modalities, including the service robot.

All of the sensor modalities that support our intelligent room are connected to our town management system (TMS) [18]. The TMS integrates the data coming from the different sensor modalities into a database that contains the information about the state of the environment. Moreover, the TMS communicates with the service robot and provides it with real-time information on its dynamically changing surroundings. The information flow between our intelligent room and the TMS is shown in Figure 14.

## 5. Experiments Section

In this section, we present several quantitative and qualitative experiments showing the capabilities of the informationally structured room and our humanoid service robots to solve different situations. We first show the performance of our tracking system for the service trolley and the wheelchair. Then, we present results showing the performance of our 3D object categorization system. Finally, we present two different scenarios in which the informationally structured room interacts with our service robot.

### 5.1. Tracking of Movable Furniture

**Figure 15:**
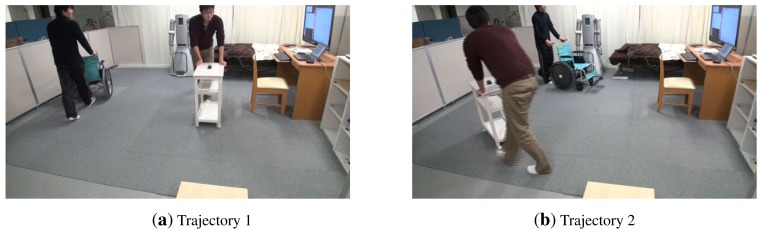
The service trolley and the wheelchair are moved simultaneously inside the room in two different trajectories (**a**) and (**b**).

In the following experiment, we show the performance of our tracking system for non-static pieces of furniture. In particular, we show results for the tracking of the service trolley and wheelchair used in our room. These objects are shown in Figure 4. In this experiment, we move the service trolley and the wheelchair in the middle of the room in circles. Both objects where moved simultaneously inside the room, as shown in Figure 15. In this way, we also want to check the robustness our different object detectors.

**Figure 16:**
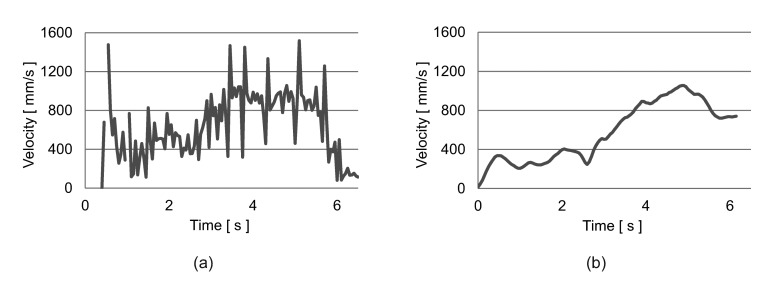
A comparison of the velocity measured by our floor sensing system (**a**) and the inertial sensor (**b**) for the service trolley.

**Figure 17:**
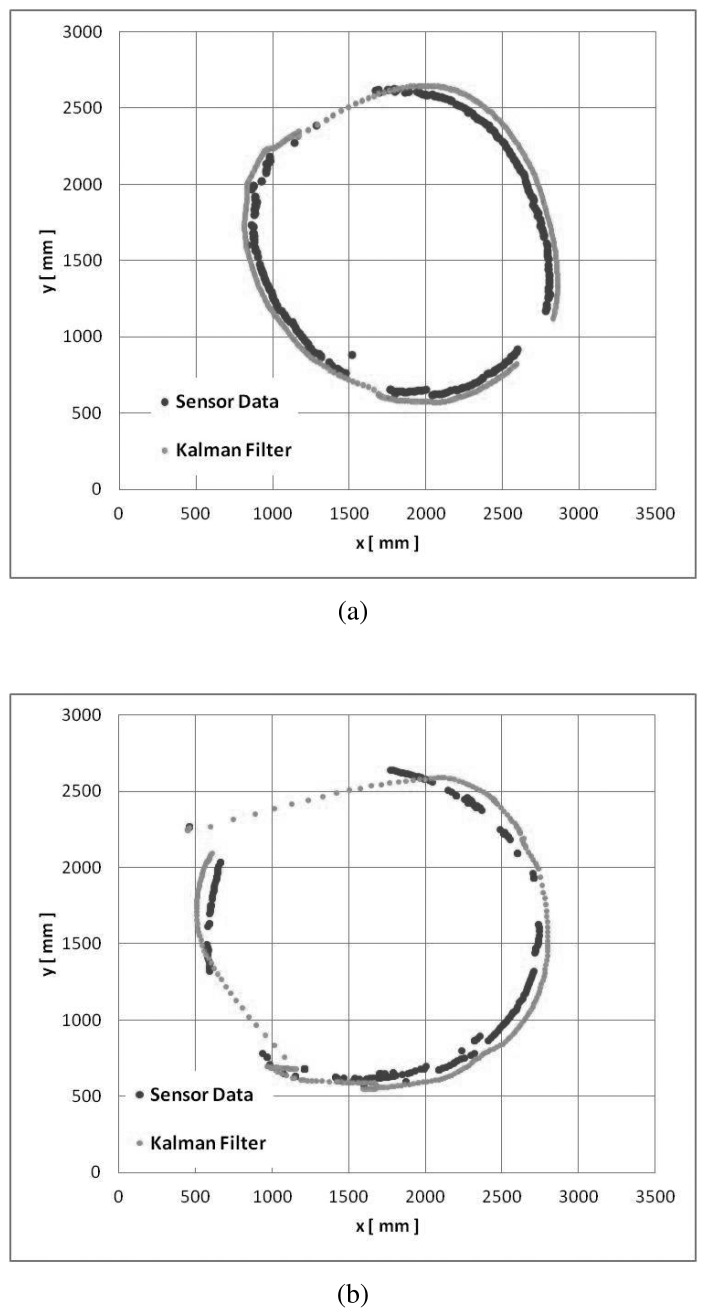
(**a**) image shows the trajectory calculated by our sensor system in comparison to the corrected trajectory using a Kalman filter for the service trolley; (**b**) depicts the same information for the wheelchair.

In Figure 16, we can see a comparison between the velocity estimated by the floor sensing system and the velocity estimated by the inertia sensor for the service trolley. These two velocity profiles are correlated and used to detect the service trolley. A similar approach is used for the wheelchair. We further apply a Kalman filter to improve the tracking of the movable furniture. Figure 17 shows the final tracking results for the trolley and the wheelchair.

### 5.2. Single Person Identification

In this experiment, we verify the identification of persons using our intelligent slippers. For this, we have captured the gate motions of 12 persons. The auto-regressive parameters for our models are calculated using eight steps, and the final feature vector is obtained by normalizing the parameters. To detect a new person, we compare the new obtained data with the calculated model for each person, and a minimum distance criterion is applied.

To test our system, we performed cross-validation 10,000 times by changing the training and test data randomly. The average success rate was 76.0% with a standard deviation of 4.5%.

### 5.3. Tracking of Multiple Persons

**Figure 18:**
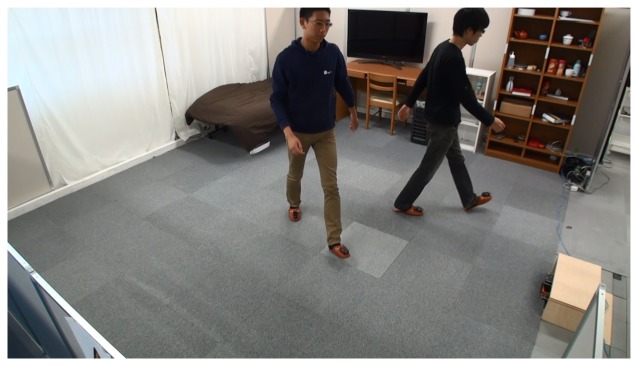
This image shows one shot of multiple person tracking.

**Figure 19:**
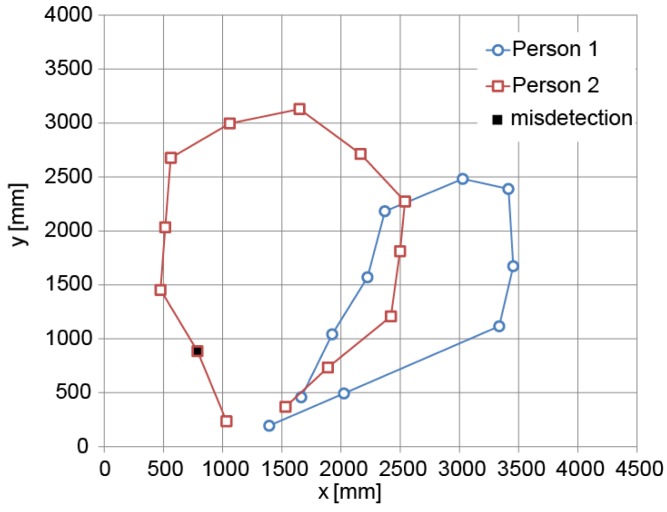
Feet detection for the two persons inside the room.

To show the performance of our tracking system for multiple persons, we carried out an experiment in which two persons walked simultaneously inside the room, as shown in Figure 18. The detection of each person is depicted in Figure 19. Most of the feet are correctly detected for each person. The detection rate was 96% in this case.

### 5.4. Categorization of New and Moved Detected Objects

In the following experiments, we present quantitative and qualitative results for the visual memory system used by the service humanoid robot working in the intelligent room. For each individual experiment, the robot took two observations of the same scene from different viewpoints. Then, after applying the change detection approach described in Section 3.1, we obtained the clusters corresponding to new or moved objects in the scene. These objects were then categorized into the different categories in our dataset (see Figure 12). We repeated the experiments in different scenarios by changing the tables and the objects on them.

As explained in Section 3.1, we compared two different methods to calculate the distance of the best matching one. Table 2 shows the results of both methods using our dataset of objects. For this experiment, we divided our dataset of objects (see Section 3.1) into random training and test sets, and we tested the different distance measures.

**Figure 20:**
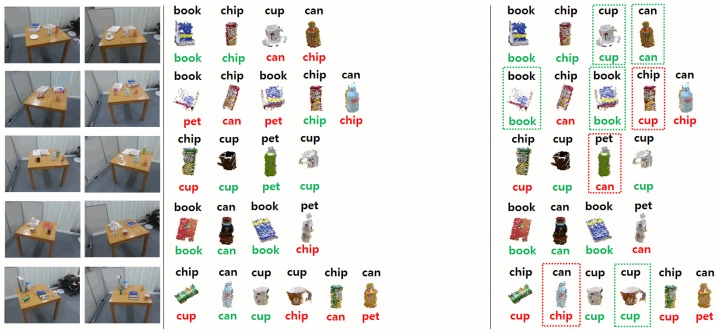
Resulting matching of changes on different tables and desks. For each scene, the two images on the left show the different views obtained by the robot. The third and fourth columns depict the categorization of the clusters corresponding to changes using method 1 (third column) and method 2 (fourth column). Below each cluster, we can see the categorization of our system. Green indicates correct categorization, and red indicates an error. Differences in the categorization between the two methods are indicated by a rectangle around the corresponding detected objects.

**Figure 21:**
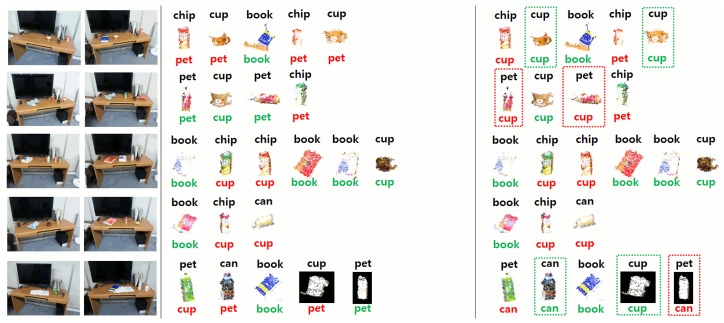
Resulting matching of changes on different tables and desks. For each scene, the two images on the left show the different views obtained by the robot. The third and fourth columns depict the categorization of the clusters corresponding to changes using method 1 (third column) and method 2 (fourth column). Below each cluster, we can see the categorization of our system. Green indicates correct categorization, and red indicates an error. Differences in the categorization between the two methods are indicated by a rectangle around the corresponding detected objects.

**Figure 22:**
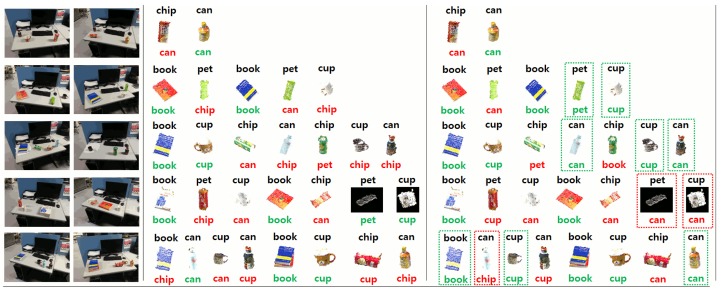
Resulting matching of changes on different tables and desks. For each scene, the two images on the left show the different views obtained by the robot. The third and fourth columns depict the categorization of the clusters corresponding to changes using method 1 (third column) and method 2 (fourth column). Below each cluster, we can see the categorization of our system. Green indicates correct categorization, and red indicates an error. Differences in the categorization between the two methods are indicated by a rectangle around the corresponding detected objects.

In addition, we show in Figures 20–22 an example of the detection and categorization results in different real situations inside our intelligent room.

## 6. Scenarios

In this section, we present different working scenarios in which our service robot interacts with the user inside the intelligent room. These scenarios are aimed at presenting qualitative and quantitative results and to show a complete view of our integrated system.

### 6.1. Simultaneous People and Object Detection

In our first scenario, our system aims to detect people together with the objects with which they are interacting.

The scenario is presented as follows.

1. Initially, Object_1is placed inside one of the intelligent cabinets, and Object_2 is placed on the table.
2. Person_1 enters the room and sits on the bed.
3. Person_2 enters the room and goes to the intelligent cabinet.
4. Person_2 takes Object_1 from the intelligent cabinet and puts it on the table.
5. Person_2 leaves the room.
6. Person_1 approaches table and takes Object_1 and Object_2.
7. Person_1 puts Object_1 and Object_2 inside the intelligent cabinet.
8. Person_2 leaves the room.

**Figure 23:**
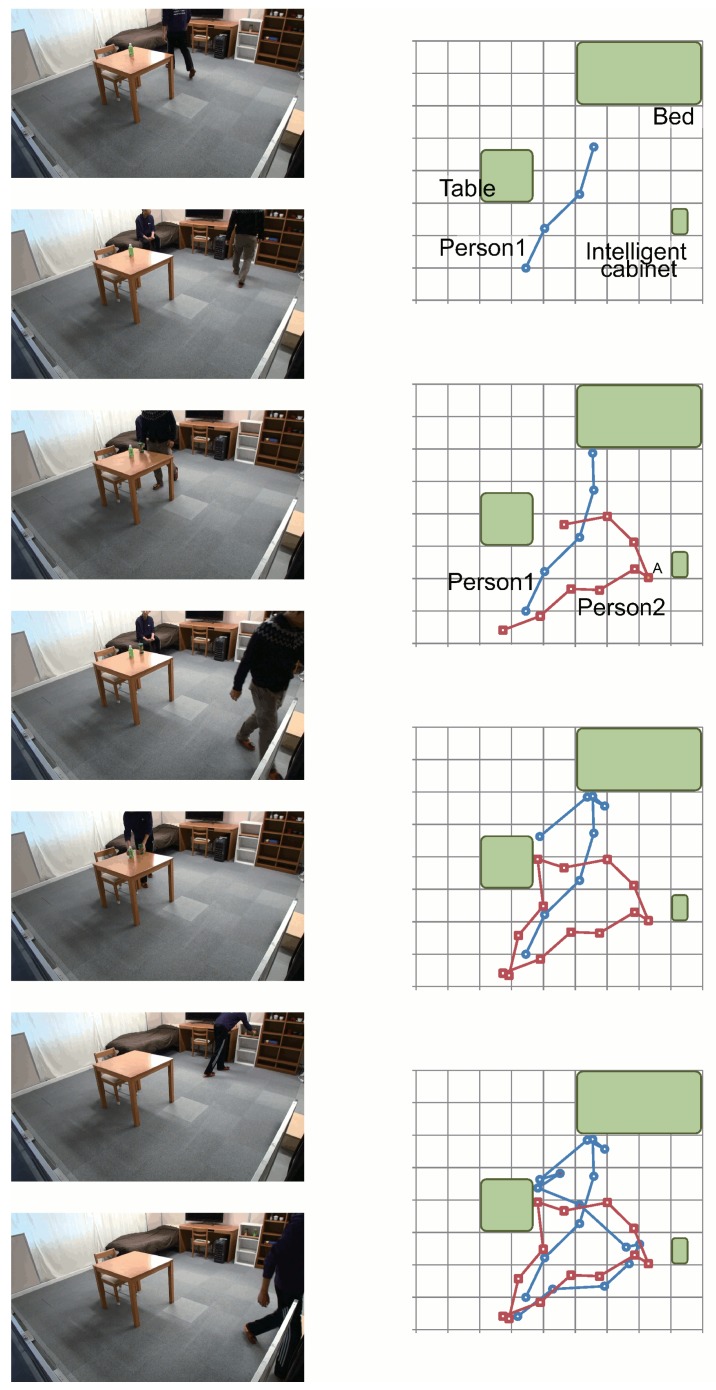
Tracking persons and objects.

The different situations in this scenario are shown in Figure 23. Snapshots of our database with the corresponding timestamps are shown in Table 3. Data corresponding to the latest timestamp are shown first. Initially (timestamp = 15:39:06), Object_2 (ID = 54) is located outside of the cabinet (state = 0). Then (timestamp = 15:40:09), Object_1 (id = 53) is located in the intelligent cabinet (ID = 15, state = 1), and the corresponding position and weight inside the cabinet are recorded. At 15:42:05, Object_1 is removed from the cabinet. The floor sensing system detected that Person_2 stood near the intelligent cabinet during 15:42:04–15:42:06. Then, the object tracker estimated that Person_2 moved Object_1. Later, Object_1 is detected again at 15:42:20, and Object_2 is detected at 15:42:21 by the cabinet. The system successfully estimates movements of objects and persons.

### 6.2. Finding Moved Objects

In this experiment, we show qualitative results demonstrating the capability of our system to find the position of an object that was moved by a person. This scenario makes use of several sensor modalities inside the intelligent room, in particular the intelligent cabinets, the floor sensing system and the visual memory of the robot. The idea of this experiment is to show how the room can update the position of objects to make them available in the future to the inhabitant or to the robot.

The sequence of actions carried out by the person inside the room are as follows:

1. A person enters the room and picks up a beverage from the intelligent cabinet.
2. This person walks around the room close to the bed, desk and table.
3. Finally, the person drinks the beverage in front of the table and leaves the bottle on the table.
4. Leave the bottle at the table.
5. Exit from the room.

Meanwhile, the person is acting in our intelligent room as follows:

1. The floor system starts tracking the person after the bottle is taken from the intelligent cabinet.
2. The different pieces of furniture are assigned a “residence time”, which is the time a person spends close to it.
3. After the person leaves the room, the system orders the different pieces of furniture according to their residence time.
4. The piece of furniture with the highest residence time is selected (the table in this case).
5. The robot approaches the table and identifies the cluster corresponding to the change (bottle).
6. The robot identifies the commodity using the RFID tag and updates its position in the database.
7. If the object is not found in the current piece of furniture, then we continue with the next one having the highest residence time.

**Figure 24:**
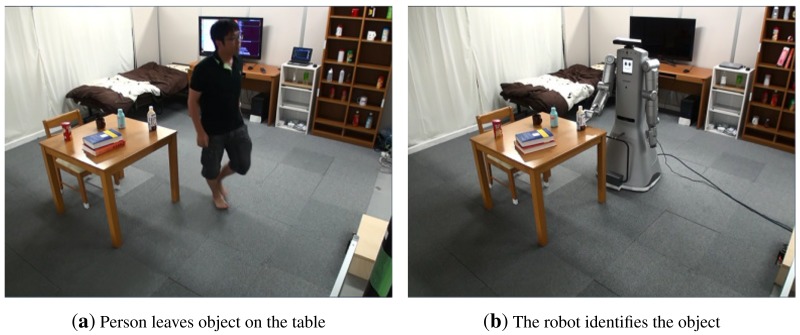
In (**a**) the habitant in the room leaves an object on the table; In (**b**) the robot identifies the object with the RFID sensors located on its hands.

Some snapshots of the previous scenario are shown in Figure 24. A full video with the experiment is available at [40].

### 6.3. Go-And-Fetch Task

In this scenario, our service robot serves a beverage from the service trolley to the person on the bed. The different steps in this situation are as follows:

1. A person enters the room pushing the service trolley.
2. The floor sensing system identifies and measures the position of the service trolley.
3. The person on the bed asks the robot to go and fetch the beverage on the service trolley.
4. The robot approaches the wagon and grasps the object from the service trolley.
5. The service robot hands over the object to the person on the bed.

**Figure 25:**
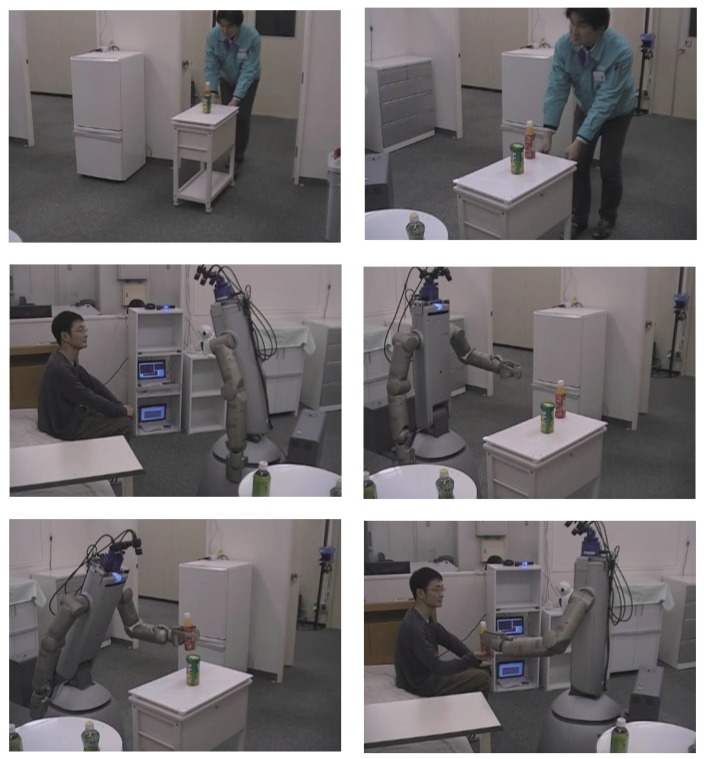
Different snapshots showing our service robot serving a beverage.

Different snapshots of the previous situation are shown in Figure 25. In this previous scenario, the position of the wagon is determined using the floor sensing system and inertial sensor, as explained in Section 2.6. Moreover, the object position is determined with the tag reader and force sensor on the service trolley. The robot grasps the object by using our planning method [41]. In this case, we have substituted the Kinect camera by a three-camera stereo system [42]. This demonstration is supported by the NEDOIntelligent Robotic Technology (RT)Software Project. Several modules, such as voice recognition and stereo vision, are connected to our system. A full video with the experiment is available at [40].

## 7. Conclusions

In this paper, we have introduced our informationally structured room, which is designed to support daily life activities of elderly people. Our room is equipped with different sensor modalities that are naturally integrated in the environment, reducing the invasion of the personal space of the habitant. In addition, the room is supported by a humanoid robot, which uses the information of the environment to assist the habitants of the room.

Privacy is an important concern in our our work, and for this reason, our room does not contain sensors that can invade the private life of people. We do not use vision cameras to track people activity; in contrast, we use the laser range finder of our floor sensing system and the IMUs attached to the slippers. The humanoid robot is equipped with an RGB-D camera, but its use is restricted to object detection and manipulation. Nevertheless, information about people activity is recorded in the TMS system, and that can present ethical problems. However, the TMS is local to the room and does not need external communication, thus reducing the risk of propagating personal information.

In this work, we have concentrated on the go-and-fetch task, which we prognosticate to be one of the most demanding tasks for the elderly in their daily life. In that respect, we have presented the different subsystems that are implicated in this task and have shown several scenarios to demonstrate the suitability of the different sensor modalities that are used in our room.

In the future, we aim to design and prepare a long-term experiment in which we can test the complete system for longer periods of time and more complex situations.

## Figures and Tables

**Table 1:** Comparison of intelligent cabinets.

**Measurement Item**		**Proposed System**	**Previous System [19]**
Maximum response time (ms)		390	610

Position	average error (mm)	16.4	7.2
	maximum error (mm)	19	13

Weight	average error (g)	28.5	31.0
	maximum error (g)	33	55
	measurable minimum value (g)	54	54

**Table 2:** Classification results for the object categorization problem in our commodities dataset using the two methods introduced in Section 3.1.

**Method 1**					

**%**	**Chipstar**	**Book**	**Cup**	**Can**	**Pet Bottle**
Chipstar	95.83	0.00	2.08	0.00	2.08
Book	0.00	100.00	0.00	0.00	0.00
Cup	10.42	0.00	58.33	10.42	20.83
Can	6.25	0.00	12.50	72.92	8.33
Pet bottle	10.42	0.00	6.25	4.17	79.17

**Method 2**					

**%**	**Chipstar**	**Book**	**Cup**	**Can**	**Pet Bottle**

Chipstar	100.00	0.00	0.00	0.00	0.00
Book	0.00	100.00	0.00	0.00	0.00
Cup	14.58	0.00	66.67	6.25	12.50
Can	6.25	0.00	6.25	79.17	8.33
Pet bottle	10.42	2.08	16.67	0.00	70.83

**Table 3:** Object database.

**Time Stamp**	**id**	**x**	**y**	**z**	**Weight**	**State**	**Place**	**Event**
15:42:21.978	54	298.706	124.350	620	343	1	15	object2 detected
15:42:20.032	53	94.6918	122.113	620	159	1	15	object1 detected
15:42:05.778	53	NULL	NULL	NULL	166	0	0	object1 lost
15:40:09.115	53	199.723	116.265	620	166	1	15	object1 detected
15:39:06.223	54	NULL	NULL	NULL	322	0	0	object2 lost
